# Isoprocurcumenol Supports Keratinocyte Growth and Survival through Epidermal Growth Factor Receptor Activation

**DOI:** 10.3390/ijms222212579

**Published:** 2021-11-22

**Authors:** Paul Kwangho Kwon, Sung Wook Kim, Ranjit De, Sung Woo Jeong, Kyong-Tai Kim

**Affiliations:** 1 Research Institute of Industrial Science and Technology, Pohang 37673, Gyeongbuk, Korea; paul0925@rist.re.kr (P.K.K.); swjeong2014@rist.re.kr (S.W.J.); 2 Department of Life Sciences, Pohang University of Science and Technology, Pohang 37673, Gyeongbuk, Korea; kimsw@postech.ac.kr (S.W.K.); deranjit@postech.ac.kr (R.D.); 3 Division of Integrative Biosciences and Biotechnology, Pohang University of Science and Technology, Pohang 37673, Gyeongbuk, Korea

**Keywords:** EGFR signaling, natural compound, isoprocurcumenol, keratinocyte, wound healing

## Abstract

Although proliferation of keratinocytes, a major type of skin cells, is a key factor in maintaining the function of skin, their ability to proliferate tends to diminish with age. To solve such a problem, researchers in medical and skin cosmetic fields have tried to utilize epidermal growth factor (EGF), but achieved limited success. Therefore, a small natural compound that can mimic the activity of EGF is highly desired in both medical and cosmetic fields. Here, using the modified biosensor system, we observed that natural small-compound isoprocurcumenol, which is a terpenoid molecule derived from turmeric, can activate EGFR signaling. It increased the phosphorylation of ERK and AKT, and upregulated the expression of genes related to cell growth and proliferation, such as *c-myc*, *c-jun*, *c-fos*, and *egr-1*. In addition, isoprocurcumenol induced the proliferation of keratinocytes in both physical and UVB-induced cellular damage, indicative of its function in skin regeneration. These findings reveal that EGF-like isoprocurcumenol promotes the proliferation of keratinocytes and further suggest its potential as an ingredient for medical and cosmetics use.

## 1. Introduction

Skin is our body’s largest organ and provides the human body with protection from the environment [1]. It protects us from physical contact, extreme temperatures, and even sunlight. However, its ability to protect our body diminishes as the regeneration capacity and suppleness of the skin is lost due to aging [2,3]. This reduction in regeneration capability of skin is the result of decreased proliferation of keratinocytes, the primary cell type of the epidermis [4]. Thus, there have been many attempts to increase the regeneration capacity of skin by inducing the proliferation of keratinocytes [5,6].

As its name suggests, the epidermal growth factor receptor (EGFR) pathway is vital in promoting the growth, proliferation, and survival of keratinocytes [7,8,9]. The activation of the EGFR signaling pathway by epidermal growth factor (EGF) involves the activation of mitogen-activated protein kinase (MAPK)/extracellular signal-regulated kinase (ERK) and phosphoinositide 3-kinases (PI3K)/protein kinase B (AKT), which further promote the expression of genes involved in cell growth and survival [10,11]. Previous studies revealed that activation of the EGFR signaling pathway by EGF could provide positive cutaneous wound-healing and anti-aging effects [12,13,14]. Although EGF seems to be an attractive molecule for both therapeutic and skin cosmetic applications, there are several hurdles to overcome before its usage can be commercialized. Firstly, due to the problem with low yield of EGF from human sources, protein synthesis and purification of EGF is very expensive [15]. Additionally, since EGF is a protein, it is very difficult to maintain and control its activity [16]. The size of EGF, which exceeds 6000 Dalton, prevents it from penetrating the skin. According to the 500 Dalton rule, compounds larger than 500 Dalton cannot pass the corneal layer of the skin, and EGF is about 12 times larger [17]. Therefore, a small molecule that can mimic the activity of EGF may be useful in both therapeutic and the skin cosmetic fields.

*Curcuma longa* L., commonly named turmeric, is widely used as a spice and medicinal ingredient [18]. Turmeric is known as a medicinal plant that shows hypoglycemic, anti-inflammatory, and neuroprotective effects [19,20,21]. According to previous studies, turmeric is considered to have great pharmacological efficacy due to its terpenoids component, a secondary metabolite of plants [22,23]. Interestingly, in attempts to find EGF-like molecules, we came across the turmeric-derived terpenoid molecule, isoprocurcumenol (IPC), which mimics the activity of EGF and activates the EGFR signaling pathway. Although different terpenoids are widely used as a raw material for pharmaceuticals and cosmetics, the physiological activities and properties of IPC remain unclear. In this study, we demonstrate a novel physiological property of turmeric-derived IPC in the growth and survival of keratinocytes, and assess its potential as a raw material for pharmaceuticals and cosmetics.

## 2. Results

### 2.1. Recognizing Isoprocurcumenol as an EGF Analogue

**Figure 1:**
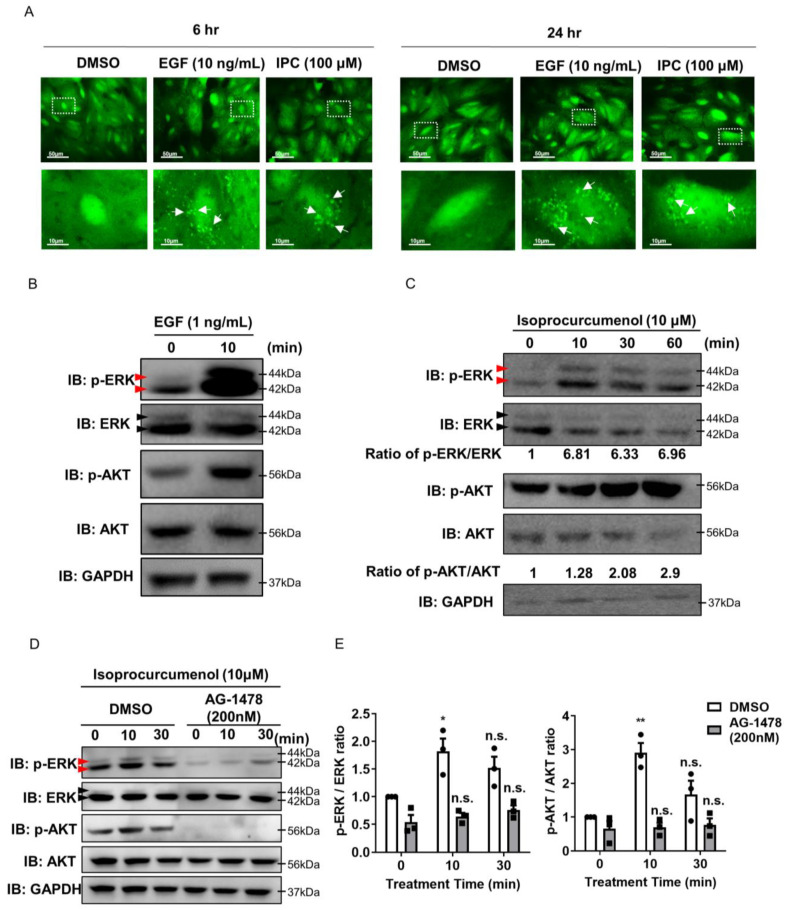
Recognizing isoprocurcumenol as an EGF analogue, using a biosensor system that measures EGFR activity. (**A**) A549 cells that stably express a biosensor system for EGFR activity were used to observe IPC as an EGF analogue. Treatment with IPC for 6 or 24 h induced GFP spots in modified A549 cells. The GFP spots are indicated with white arrows. DMSO was used as a negative control, while EGF (10 ng/mL) was used as a positive control. (**B**) Immunoblot of p-ERK and p-AKT after EGF treatment. HaCaT cells were treated with EGF (1 ng/mL) for 10 min and were subjected to Western blot analysis. Total ERK and AKT were used for normalization. Red arrows indicate phosphorylated ERK 42/44, while black arrows indicate total ERK 42/44. (**C**) Immunoblot of p-ERK and p-AKT after IPC treatment. HaCaT cells were treated with IPC for 10, 30, or 60 min and were subjected to Western blot analysis. Total ERK and AKT were used for normalization. Red arrows indicate phosphorylated ERK 42/44, while black arrows indicate total ERK 42/44. (**D**,**E**) Representative immunoblotting (**D**) and the quantification (**E**) of IPC-treated p-ERK and p-AKT after the inhibition of EGFR signaling. HaCaT cells were treated with DMSO or AG-1478, an EGFR antagonist, before treatment with IPC. Total ERK and AKT were used for normalization. Red arrows indicate phosphorylated ERK 42/44, while black arrows indicate total ERK 42/44 (*n* = 3). n.s., not significant, * *p* < 0.05, ** *p* < 0.01; unpaired Student’s *t* test; error bars indicate SEM.

To explore the analogues of EGF, we utilized the A549 cell line that stably expresses previously modified plasmid, which contains two SH2 domains fused to EGFP and EF1α promoter [24]. The system has been previously set up to explore small molecules that could activate EGF receptor (EGFR). The plasmid allows the cell to show spot-like GFP signals when treated by EGF, as the activated EGFR is endocytosed into the cell. Through this system, we screened for possible EGFR activators by observing whether the drug candidate induced the spot-like GFP signal in the cytoplasm. We observed the GFP spots at 6 and 24 h after drug treatment. Through screening of multiple drug libraries, we found that isoprocurcumenol (IPC) could activate EGFR. When we treated EGF as the positive control, we were able to observe strong spot-like GFP signals in the cell (Figure 1A). Similar to EGF, IPC could induce strong spot-like GFP signals in cells 6 and 24 h after treatment, which indicates the activation of EGFR by IPC (Figure 1A and Figure S1A). Although some other compounds activate EGFR, we focused on IPC because it internalized greatest number of GFP spots similar to the effect of EGF (Figure S1B).

To confirm whether IPC can activate EGFR, we measured the level of proteins located downstream of EGFR signaling. ERK and AKT are well-known protein kinases that are regulated by EGFR signaling [10]. Both ERK and AKT are known to be involved in the growth, proliferation, and survival of the cell, which is similar to the function of EGF [11]. Due to the fact that previous reports observed increases in the phosphorylation of ERK and AKT by treatment with EGF [24], we investigated whether IPC could also induce the phosphorylation of both ERK and AKT. From this point on, we used the human keratinocyte cell, HaCaT cell, for all experiments. We first observed that EGF treatment induced phosphorylation of ERK and AKT, similar to the results obtained in previous studies (Figure 1B). Analogous to EGF, we observed the phosphorylation of ERK and AKT by IPC treatment (Figure 1C). IPC seemed to induce the phosphorylation of ERK and AKT after 10 min and this was sustained for 1 h (Figure 1C). To confirm whether the phosphorylation of ERK and AKT by IPC were through EGFR signaling, we treated the cells with the EGFR antagonist, AG1478, prior to IPC treatment (Figure 1D). The phosphorylation of ERK and AKT did not increase significantly by IPC treatment in the presence of AG1478 (Figure 1E). These data confirm that the increased phosphorylation of ERK and AKT induced by IPC was through EGFR signaling, and that IPC is an analogue of EGF.

### 2.2. Measuring Appropriate Concentration of Isoprocurcumenol to Induce Cell Proliferation

**Figure 2:**
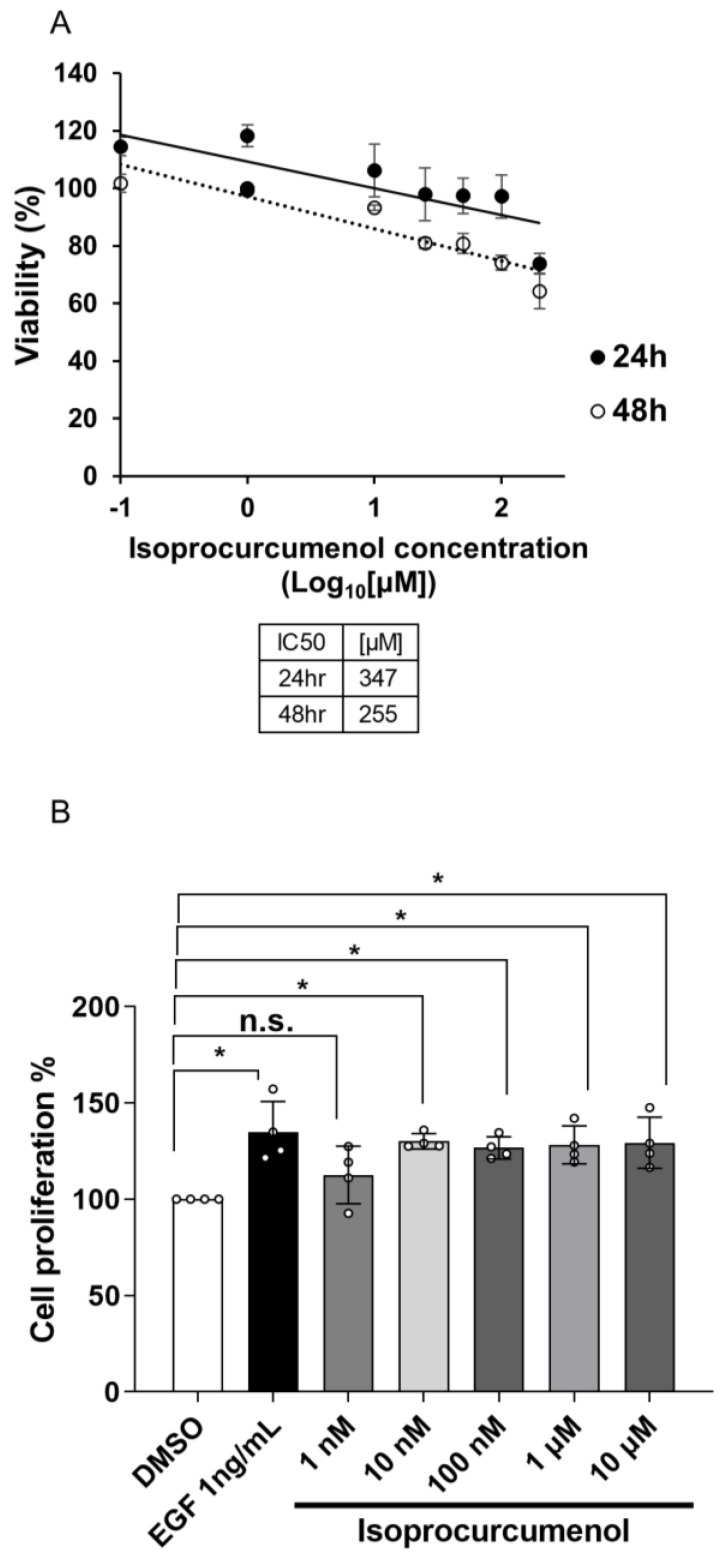
Measuring the concentrations of isoprocurcumenol that induce cell proliferation. (**A**) Measuring the toxicity of IPC on HaCaT cells. HaCaT cells were treated with different concentrations of IPC (0 nM, 100 nM, 1 μM, 10 μM, 25 μM, 50 μM, 100 μM, or 200 μM) for 24 or 48 h. MTT assay was performed to measure the viability of HaCaT cells. DMSO was used as a negative control (*n* = 4). unpaired Student’s *t* test; error bars indicate SEM. (**B**) Measuring the proliferation of HaCaT cells induced by IPC. HaCaT cells were treated with different concentrations of IPC (1 nM, 10 nM, 100 nM, 1 μM, or 10 μM) for 24 h. Cell Counting Kit-8 (CCK-8) was used to measure the proliferation of IPC-treated HaCaT cells. DMSO was used as a negative control, while EGF (1 ng/mL) was used as a positive control (*n* = 4). n.s., not significant, * *p* < 0.05; one-way ANOVA; error bars indicate SEM.

In order to use IPC as an analogue of EGF, we had to confirm its toxicity on cells by measuring cell viability through MTT assay. According to ISO 10993-5, a guideline for biological evaluation of in vitro cytotoxicity from the International Organization of Standardization, drugs that induce 30% or greater decrease in cell viability are known to have cytotoxicity. In the case of IPC, its treatment at a concentration of 200 μM for 48 h showed cytotoxicity, as it induced a 40% decrease in cell viability (Figure 2A). This means that IPC with concentrations less than 200 μM will be safe to use. We also calculated the half-maximal inhibitory concentration (IC50) for IPC and found it to be 347 μM at 24 h and 255 μM at 48 h of treatment (Figure 2A).

We then measured the minimum concentration of IPC that can induce cell proliferation through CCK-8 assay. There was a significant increase in the proliferation of cells at most of the IPC concentrations, starting at 10 nM. Only the IPC with 1 nM of concentration failed to increase cell proliferation significantly (Figure 2B). These data show that IPC is an EGF-like molecule that can safely induce cell proliferation at low concentrations.

### 2.3. IPC Induces the Expression of Genes Related to Cell Growth and Proliferation

**Figure 3:**
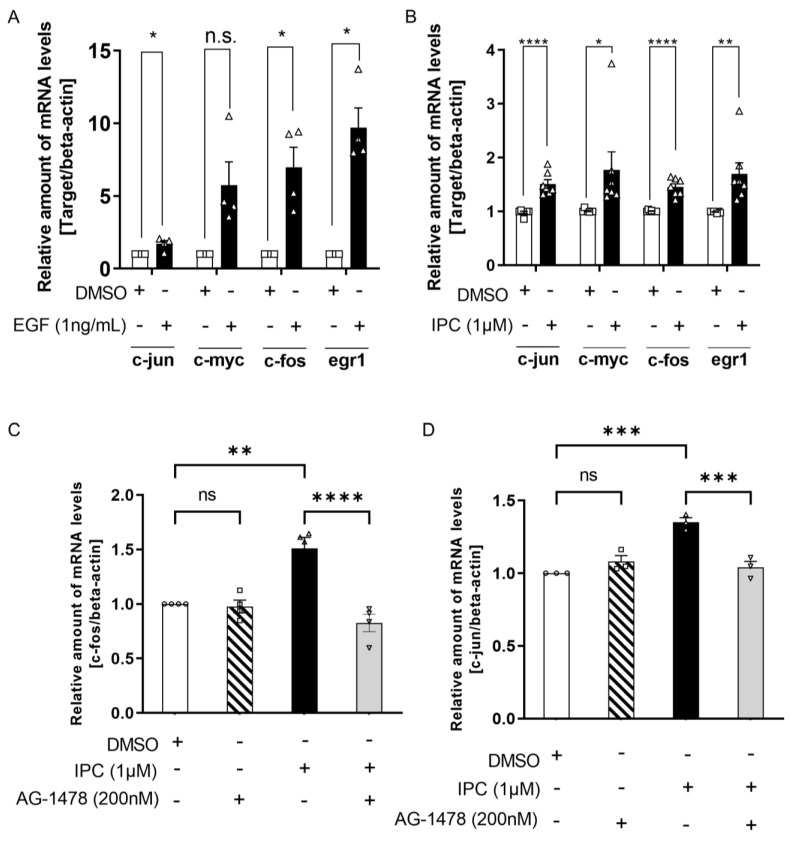
IPC induces the expression of genes related to cell growth and proliferation. (**A**) Measuring the effect of EGF on mRNA level of genes related to cell growth and proliferation. HaCaT cells were treated with EGF (1 ng/mL) for 1 h. Total RNA extracted from HaCaT cells were reverse-transcribed into cDNA, and were subjected to real-time quantitative polymerase chain reaction (RT-qPCR). mRNA levels of *c-jun*, *c-myc*, *c-fos*, and *egr1* were measured. mRNA level of *beta-actin* was used for normalization (*n* = 4). n.s., not significant, * *p* < 0.05; unpaired Student’s *t* test; error bars indicate SEM. (**B**) Measuring the effect of IPC (1 μM) on mRNA level of genes related to cell growth and proliferation. HaCaT cells were treated with IPC for 1 h. Similarly to (**A**), mRNA levels of *c-jun*, *c-myc*, *c-fos*, and *egr1* were measured. The mRNA level of *beta-actin* was used for normalization (*n* = 4). n.s., not significant, * *p* < 0.05, ** *p* < 0.01, **** *p* < 0.0001; unpaired Student’s *t* test; error bars indicate SEM. (**C**,**D**) Measuring the effect of IPC on *c-fos* (**C**) and *c-jun* (**D**) mRNA levels after blocking EGFR signaling. HaCaT cells were treated with AG-1478, an EGFR antagonist, before being treated with IPC. mRNA levels of *c-fos* and *c-jun* were measured through RT-qPCR. The mRNA level of *beta-actin* was used for normalization. The experiment was repeated four times for (**C**) and three times for (**D**). n.s., not significant, ** *p* < 0.01, *** *p* < 0.001, **** *p* < 0.0001; ordinary one-way analysis of variance (ANOVA) with Tukey’s multiple comparison test; error bars indicate SEM.

Previous results have confirmed that IPC induced cell growth and proliferation, similar to the effect of EGF, so we sought to identify the mechanism of such effect. According to previous studies, genes like *C-myc*, *C-jun*, *C-fos*, and *Egr-1* are well associated with cell growth and proliferation. Hence, we assessed whether the treatment of IPC can induce the expression of these aforementioned genes. We measured the mRNA level of *c-myc*, *c-jun*, *c-fos*, and *egr-1* after treatment with either EGF or IPC by performing real-time quantitative polymerase chain reaction (RT-qPCR). As expected, mRNA levels of *c-jun*, *c-fos*, and *egr-1* were significantly increased by treatment with EGF (Figure 3A). However, the mRNA level of *c-myc* was increased but showed no significance (Figure 3A). When we treated IPC, the mRNA levels of all four genes were significantly increased (Figure 3B).

From there on, we determined whether the increases in the mRNA levels of *c-fos* and *c-jun* by IPC were due to the activation of EGFR signaling. We treated the cells with the EGFR antagonist, AG1478, before IPC treatment and measured the mRNA level of *c-fos* and *c-jun.* Similar to the results obtained for EGF (Figure S2A,B), increased mRNA levels of *c-fos* and *c-jun* by IPC treatment were no longer seen after treatment with AG1478 (Figure 3C,D). This indicates that the effect of IPC on the expression of genes related to cell growth and proliferation was through EGFR signaling. Collectively, IPC induced growth and proliferation of the cell by increasing the expression of genes, such as *c-fos*, *c-jun*, *c-myc*, and *egr-1,* through activation of the EGFR signaling pathway.

### 2.4. IPC Induces Cell Recovery and Wound Healing

**Figure 4:**
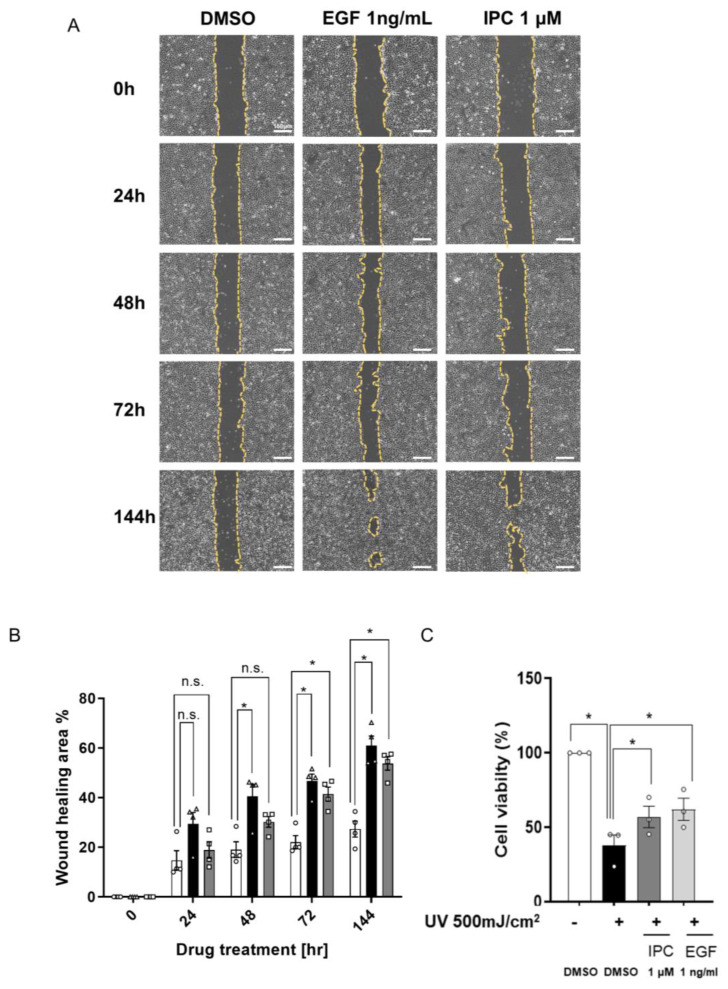
The effect of IPC on cell recovery and wound healing. (**A**,**B**) Representative image (**A**) and the quantification (**B**) of wound healing effect of IPC. Wounds were generated by scratching the bottom of the plate with a pipette tip. Cells were incubated with IPC for up to 144 h. The area of the wounds, indicated by the yellow dotted line, were measured at each indicated time point to observe the wound healing effect. DMSO was used as a negative control, while EGF was used as a positive control. The color of bar graph indicates following conditions: white—DMSO, black—EGF, and gray—IPC (*n* = 4). n.s., not significant, * *p* < 0.05; unpaired Student’s *t* test; error bars indicate SEM. (**C**) Measuring the cell recovery effect of IPC after UVB damage. HaCaT cells were exposed to UVB (500 mJ/cm^2^) before being treated with either DMSO, EGF, or IPC. CCK-8 was used to measure the viability of UBV-damaged HaCaT cells. DMSO was used as a negative control, while EGF was used as a positive control (*n* = 3). n.s., not significant, * *p* < 0.05; unpaired Student’s *t* test; error bars indicate SEM.

Through previous experiments, we examined the effect of IPC on cell proliferation and found that it can safely induce cell proliferation at low concentrations. We also observed that IPC increased the expression of genes related to cell proliferation, such as *c-fos* and *c-jun*. This led us to assume that IPC might have an effect on cell recovery, similar to EGF. Thus, to confirm the effect of IPC on cell recovery and migration, wound healing assay was performed [24]. We treated the physical wound with EGF or IPC and investigated their effects on wound healing. EGF is known for its wound healing effect, hence, the wound was significantly healed at 48 h after treatment and was almost completely healed 144 h after treatment (Figure 4B). Similar to EGF, IPC also has a wound healing effect. The area of the wound was significantly reduced starting at 72 h after treatment (Figure 4B). This shows that IPC has wound healing effect on cells, just like EGF.

However, there are other factors, aside from physical damage, that adversely affect cells and the skin. An environmental factor, like strong UV light, can surely damage the skin [25]. Hence, we demonstrated whether the treatment with IPC can maintain the viability of cells with ultraviolet B (UVB)-induced damage (Figure S3). Decreased cell viability by UVB damage was significantly recovered by EGF treatment (Figure 4C). Similar to EGF, IPC treatment also induced a significant recovery of cell viability (Figure 4C), confirming its wound healing effect on cells, even when cells have been damaged by UVB exposure. Conjointly, we demonstrated that IPC is an EGF-like molecule that functions in a similar way to EGF, in that it induces significant cell recovery from physical damage and maintains viability of the cell even after UVB damage.

## 3. Discussion

Epidermal growth factor (EGF) plays a significant role in the growth and proliferation of keratinocytes by activating EGFR signaling pathways [10,11]. Thus, EGF has been well known for its effect in wound healing and skin regeneration [26]. Although there have been some efforts to utilize EGF in pharmaceutical and cosmetic materials, there are various issues with the use of EGF. As mentioned previously, the synthesis and purification of EGF is very costly and it is very difficult to maintain the activity of isolated or purified EGF [16]. Therefore, a natural compound that can mimic the effect of EGF is a useful alternative material for both pharmaceutical and cosmetic fields. Many natural compounds, such as terpenoids, are found in the roots of different plants [22]. The root of turmeric has been known to possess various terpenoids that have hypoglycemic, anti-inflammatory, and neuroprotective effects [19,20,21].

Isoprocurcumenol (IPC) is one of the terpenoids found in turmeric that can be purified from the rhizomes [27,28]. Herein, we found that turmeric-derived natural compound, IPC, has an EGF-like activity. We initially observed the activation of the EGFR signaling pathway by IPC. The treatment of IPC activated both ERK and AKT through the EGFR signaling pathway, which was confirmed by treatment with the EGFR antagonist, AG-1478. We also observed a significant increase in the proliferation of keratinocytes following treatment with IPC. Additionally, the expression of genes related to cell growth and proliferation, like *c-myc*, were significantly increased by treatment with IPC. Although these results indicate a positive effect of IPC on cell growth, some may be concerned that excessive use of IPC may lead to tumorigenesis. Previous studies have shown that excessive activation of EGFR signaling can promote pathogenesis of different cancers [29,30], while its target genes such as *c-myc*, *c-fos*, and *c-jun*, are known to be proto-oncogenes [31]. Although the expression of EGF declines with age [32], the use of IPC should be regulated properly in order to avoid the pathogenesis of tumor.

We were also able to confirm the substantial wound healing effect of IPC on cells with physical damage and its effect on maintaining cell viability after UVB irradiation. These results indicate that, like EGF, IPC also has an effect on skin regeneration. Although EGF has been investigated as a wound healing agent, topically delivered EGF tends to degrade in the actual wound environment [33]. As IPC is a natural compound that tends to have higher stability as a drug [34], it would be a great replacement for EGF as a wound-healing and skin-regenerative agent. Additionally, IPC is reported to have an anti-oxidant effect [35]. Thus, increased oxidative stress and wounds caused by prolonged exposure to UVB could be alleviated by IPC alone.

Unlike previously found EGF alternatives, IPC clearly has advantages over EGF or other EGF-like molecules. The cost of IPC production is much cheaper than EGF because the source of IPC, the root of turmeric, is easier to obtain than human sources for EGF. At the time of the publication, recombinant human EGF from Roche costs 40 times more than IPC from ALB materials. In addition, the molecular weight of IPC (234.4 Dalton) is substantially less than EGF (approximately 6200 Dalton) and is clearly under the 500 Dalton rule of skin penetration [17]. Additionally, the working concentration of IPC may not be lower than EGF, but it is much lower than other known EGF-like molecules. The effective concentration of piperonylic acid, which is an EGF-like molecule that we previously found, is 100 μM [24], while the effective concentration of IPC is 1 μM. Therefore, these advantages of IPC over EGF or other EGF-like compounds show that IPC is an attractive and effective cosmetic and pharmaceutical material.

In conclusion, we confirmed the potential of IPC as a great natural EGF alternative that can be used in pharmaceutical and cosmetic products.

## 4. Material and Methods

### 4.1. Cell Culture and Compound Treatment

Modified A549 cells and HaCaT cells were cultured in Dulbecco’s modified Eagle’s medium (HyClone) supplemented with 10% fetal bovine serum (HyClone) and 1% antibiotics (WelGENE) and were maintained in a humidified incubator with 95% air and 5% CO_2_ at 37 °C. The cells were incubated in serum-free DMEM for 24 h before the treatment of compounds such as epidermal growth factor (EGF) (Peprotech), EGFR antagonist (AG1478), isoprocurcumenol (IPC) (ALB Materials), and 0.1% DMSO. We screened with a drug library that contained a total of 305 natural compounds. The library was specifically made for our use under the name of MNP-Natural Compound (Interpharm, Goyang, Korea). The fluorescence images of modified A549 cells were obtained using a ZEISS fluorescent microscope. All cell experiments were performed within 3–10 cell passages.

### 4.2. SDS-Polyacrylamide Gel Electrophoresis (PAGE) and Immunoblotting

Drug-treated cells were lysed in a cell lysis buffer (50 mM Tris pH 7.5, 150 mM NaCl, 1 mM EDTA, and 1% Triton X-100). Cell lysates from different experiments were subjected to SDS-PAGE to measure the level of protein. Loading samples were prepared with 20 or 30 μg of cell lysates mixed with 5× sample buffer (0.6% 1 M Tris, 50% glycerol, 10% SDS, 0.5% 2-mercaptoethanol, and 1% bromophenol blue). The samples were loaded onto the Western blot gel and were resolved in electrophoresis chambers (Bio-Rad). Then, the proteins in the gel were transferred to the nitrocellulose membrane (Pall Corporation) using the same power supply and transfer chamber (Bio-Rad). For immunoblotting, the membranes were incubated in *α*-p-ERK T202/Y204, *α*-ERK, *α*-p-AKT S473, *α*-AKT, or *α*-GAPDH (Millipore) primary antibodies overnight at 4 °C. Then, the membranes were incubated in horseradish peroxidase (HRP)-conjugated secondary antibodies. Finally, chemiluminescence images were obtained by ImageQuant LAS 4000 (Fuji) [36,37].

### 4.3. Cell Growth Assay

Before the experiment, 5 × 10^3^ HaCaT cells were plated on each well of a 96-well plate for 24 h. Then, the cells were cultured in serum-free DMEM for 24 h to deplete any growth factors in media. The cells were then treated with EGF, IPC, or DMSO mixed in serum-free DMEM for 24 h. Following the manufacturer’s instructions, Cell Counting Kit-8 (CCK-8, Dojindo) was used to measure the growth of the cells (OD: 450 nm).

### 4.4. Cell Viability (MTT) Assay

Before the experiment, 1 × 10^4^ HaCaT cells were plated on each well of a 96-well plate for 24 h. Then, the cells were cultured in serum-free DMEM for 24 h to deplete any growth factors in media. The cells were then treated with IPC or DMSO mixed serum-free DMEM for 24 or 48 h. After each time point, the cells were incubated in a medium that contained 3-(4,5-dimethylthiazol-2-yl)-2,5-diphenyltetrazolium bromide (MTT, 5 mg/mL) for 2 h at room temperature. Then, dimethyl sulfoxide (DMSO) was added to each well after completely removing the media. The mixture was then subjected to OD measurement at 570 nm using a NanoQuant spectrophotometer (Tecan) [38].

### 4.5. RNA Extraction and Quantitative PCR

TRI Reagent (BioScience Technology) was used to extract the total RNA from HaCaT cells as previously described [39,40,41]. According to the manufacturer’s instructions, RNA (1 μg) was reverse-transcribed using ImProm-II™ reverse transcriptase (Promega, Madison, WI, USA) to create cDNA. The cDNA levels of each target gene were measured by quantitative real-time PCR using the StepOnePlus real-time PCR system (Applied Biosystems, Waltham, MA, USA) with FastStart Universal SYBR Green Master (Roche, Basel, Switzerland). The sequences of the forward and reverse primers of *c-jun*, *egr-1*, *c-myc*, *c-fos*, and *β-actin* were described previously [24].

### 4.6. Wound Healing Assay

Before the experiment, 2 × 10^5^ HaCaT cells were plated on each well of a 12-well plate for 24 h. Then, a 200 μL standard pipette tip (Sorenson Biosciences) was used to scratch the bottom of the plate to create a wound area [24]. The cells were then cultured in IPC, EGF, or DMSO containing serum-free DMEM. Olympus phase contrast inverted microscope and ImageJ were used to obtain and quantify the images of wounded areas [42].

### 4.7. UVB Irradiation

Before the experiment, 1 × 10^4^ HaCaT cells were plated on each well of a 96-well plate for 24 h. Then, cells were washed with PBS before adding a thin layer of PBS. Cells were then exposed to UVB light (500 mJ/cm^2^) using CL-1000 (UVP, Analytik Jena, Jena, Germany). Then, cells were maintained in serum-free DMEM with DMSO, EGF, or IPC for 24 h. Control cells were maintained without UVB irradiation.

### 4.8. Statistical Analysis

All quantitative data are presented as means ± SEM. The comparison between two groups were statistically analyzed by unpaired Student’s *t* tests. Comparisons between three or more groups with one independent variable were analyzed by ordinary one-way analysis of variance (ANOVA) with Tukey’s multiple comparison test. A result of *p* < 0.05 was considered to represent significance. All experiments were repeated at least 3 times with at least 3 replicates per experiment. *p*-values greater than 0.05 were considered not significant. The significance of the statistical analysis are indicated as such: n.s., not significant, * *p* ≤ 0.05, ** *p* ≤ 0.01, *** *p* ≤ 0.001, and **** *p* ≤ 0.0001.

## Data Availability

Data is contained within the article or Supplementary Materials.

## Supplementary Materials

The following are available online at https://www.mdpi.com/article/10.3390/ijms222212579/s1.
