# Supplementary material for: Isoprocurcumenol Supports Keratinocyte Growth and Survival through Epidermal Growth Factor Receptor Activation
Source: Int J Mol Sci. 2021 Nov 22;22(22):12579. doi: 10.3390/ijms222212579 (PMC8625800; doi:10.3390/ijms222212579)
Supplement: Supplementary file 1 [file ijms-22-12579-s001.zip › ijms-1449171-supplementary.pdf]

International Journal of *Molecular Sciences*

MDPI

Supplementary Material

Isoprocurcumenol Supports Keratinocyte Growth and Survival through Epidermal Growth Factor Receptor Activation

Paul Kwangho Kwon [1,†], Sung Wook Kim [2,†], Ranjit De [2,3], Sung Woo Jeong [1] and Kyong-Tai Kim [1,2,3,*]

[1] Research Institute of Industrial Science and Technology, Pohang 37673, Gyeongbuk, Korea; paul0925@rist.re.kr (P.K.K.); swjeong2014@rist.re.kr (S.W.J.)

[2] Department of Life Sciences, Pohang University of Science and Technology, Pohang 37673, Gyeongbuk, Korea; kimsw@postech.ac.kr (S.W.K.); deranjit@postech.ac.kr (R.D.)

[3] Division of Integrative Biosciences and Biotechnology, Pohang University of Science and Technology, Pohang 37673, Gyeongbuk, Korea

* Correspondence: ktk@postech.ac.kr

† These authors contributed equally to this study

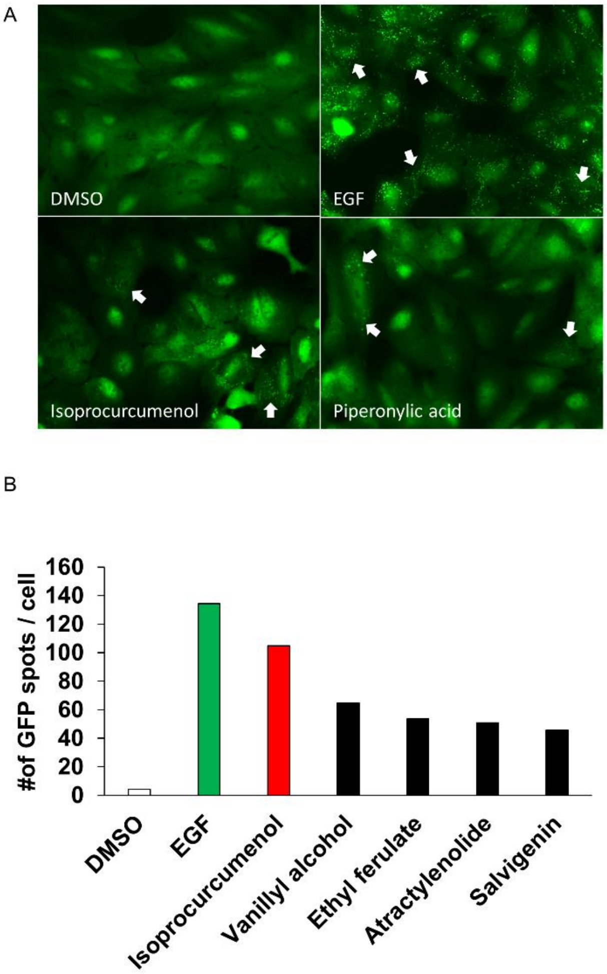

Figure S1. Comparison of IPC to other natural compounds. (**A**) Quantification of internalized GFP spots by indicated chemicals. Average number of GFP spots were counted per cell. (**B**) Comparison between IPC and piperonylic acid. Both IPC and piperonylic acid induced internalization of EGFR.

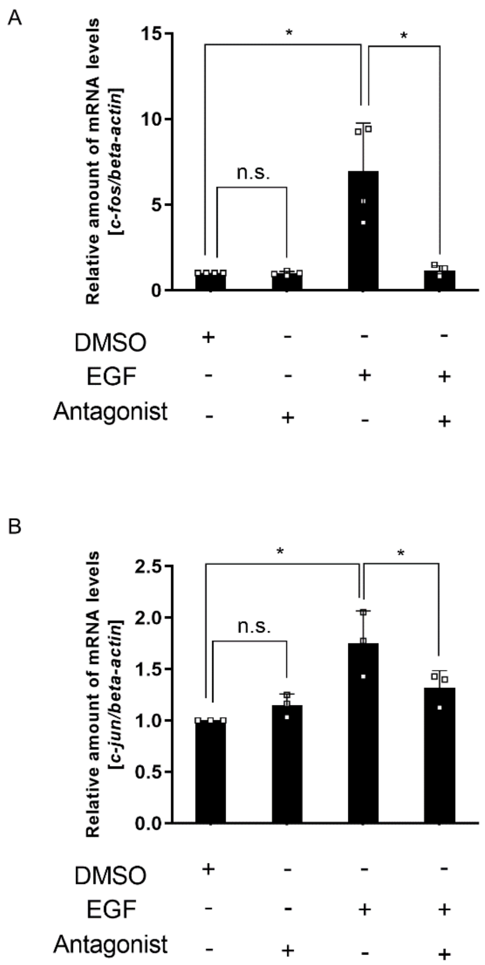

Figure S2. Measuring the effect of EGF on genes related to cell growth, after blocking EGFR signaling. (**A** and **B**) Measuring the effect of EGF on *c-fos* (**A**) and *c-jun* (**B**) mRNA level after blocking EGFR signaling. HaCaT cells were treated with AG-1478, an EGFR inhibitor, before being treated with EGF. mRNA levels of *c-fos*, and *c-jun* were measured through RT-qPCR. mRNA level of *beta-actin* was used for normalization. The experiment was repeated four times for (**A**) and three times for (**B**). n.s., not significant, *$P < 0.05$; unpaired Student's t test; error bars indicate SDs.

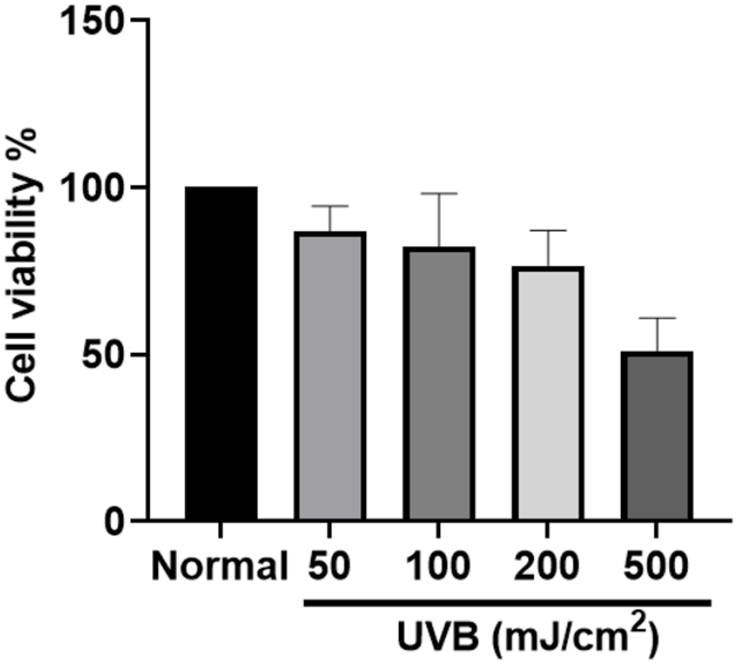

Figure S3. Finding the right condition of UV-B on cell viability**.** Cell viability was measured after UV-B irradiation. Different intensity of UV-B irradiation was treated to HaCaT cells. 500 mJ/cm^2 was strong enough to induce HaCaT cell death.
